# The Interrelationship between Family Violence, Adolescent Violence, and Adolescent Violent Victimization: An Application and Extension of the Cultural Spillover Theory in China

**DOI:** 10.3390/ijerph15020371

**Published:** 2018-02-21

**Authors:** Yiwei Xia, Spencer D. Li, Tzu-Hsuan Liu

**Affiliations:** Department of Sociology, University of Macau, Macau, 999078, China; xiayiweinemo@gmail.com (Y.X.); spencerli@umac.mo (S.D.L.)

**Keywords:** Chinese adolescents, violent behavior, violent victimization, cultural spillover theory

## Abstract

The current study is the first study to emphasize family systems, violent norms, and violent peer association as three domains of the social environment that influence both adolescent violent offending and victimization among Chinese adolescents using a longitudinal sample. Under the framework of cultural spillover theory, the purpose of the current study was to explore how these three factors influenced adolescent violent offending and victimization. A total of 1192 middle and high school students were randomly selected from one of the largest cities in Southwest China. Structural equation model analysis was applied to investigate the direct and indirect effect of violence in the family system on violent offending and victimization. The results indicated that violent offending and victimization overlapped among Chinese adolescents. Violent peer association and acceptance of the violence norm fully mediated the effect of violence in the family system on violent offending, and partially mediated the effect of violence in the family system on violent victimization. In conclusion, adolescents who had experienced violence in their family system were more likely to be exposed to violent peer influences and to accept violent norms, which increased the likelihood of violence perpetration and victimization later in their life.

## 1. Introduction

The World Health Organization (WHO) has identified violence as a thorny public health problem for decades [1]. Violence is often associated with a series of severe consequences for victims throughout their life, especially for children [2]. Children who experience violence may suffer from psychological problems, including depression, anxiety [3], and post-traumatic stress disorder (PTSD) [4], as well as display externalizing behaviors [5,6]. In addition, violence and violent behavior are shown to hinder educational and social-economic attainment [7], which are key areas of childhood development. Furthermore, the early onset of violence and victimization predicts later offending and victimization in their adulthood, described as persistence-offending [8] or circle-of-violence [9].

A significant number of children suffer exposure to violence in the family system [10]. Under the circumstances, parents may be the perpetrators and the family is no longer a safe haven for the children. Several studies on family violence have revealed that witnessing interparental violence, punitive child-rearing, and other kinds of physical abuse may engender later violent criminal behavior [11,12]. Adolescents who grow up in a violent family environment are more likely to identify violent behaviors as an acceptable way to resolve conflicts. In turn, such violent behaviors may be carried over into the school and peer relationships, resulting in a disrupted relationship with mainstream peer groups. To heighten a sense of belonging, those adolescents may join violent peer groups, learn violent norms, and commit acts of violence [13]. Association with violent peer groups that encourage violence may increase the risk of adolescents becoming a victim or a perpetrator of violence [14]. The fact that the violent culture in the family system spills over to the peer context is echoed with cultural spillover theory. According to the theory, legitimate violence, the type of violence that is socially or culturally sanctioned in specific arenas (e.g., violent sports, corporal punishment, and media violence) may spill over into unsanctioned spheres, such as relationships with intimate peers [15,16].

With exceptions, the corporal punishment of children is socially approved in China. The majority of Chinese parents physically impose discipline on their children. For example, Xing and Wang demonstrated that approximately 70% of low-grade elementary school students, and 50% of high-grade elementary school students had experienced physical punishment by using data from a nationally representative sample in China. Among those parents who imposed physical discipline on their children, 92.6% of parents imposed light physical punishment on their children, while 49.4% parents imposed severe physical punishment on their offspring [17]. Similar findings have been reported by other researchers. For example, in a study of a nationally representative sample, Tang reported that 57.5% of Hong Kong parents imposed physical discipline on their children under eighteen in the past year [18]. According to cultural spillover theory, children who experience high levels of violence in a family setting may engage in more culturally unacceptable violent behavior.

Violence and violent victimization are also severe public health issues among mainland Chinese adolescents. Despite the lack of official data, a national representative study that surveyed more than 10,000 school-aged students reported that the prevalence of lifetime single form violent victimization was 71%, while poly-victimization was 14% [19]. In terms of violence perpetration, another nation-representative study showed that the prevalence rate of perpetration among pre-college adolescents was about 9% [20]. The statistics varied markedly across regional studies or small-scale studies conducted in provinces or cities in China. The prevalence rate of victimization was found to range from 2% to 66%, whereas the rate of self-reported perpetration ranged from 2% to 34% [21].

Violence and violent victimization seldom occurs independently, instead, a considerable amount of the literature indicated that violence and violent victimization largely overlapped. After reviewing more than 37 studies that focused on offender-victim overlaps spanning a half century, Jennings et al. highlighted that there was strong evidence for the offender-victim overlaps, especially for violent behavior and victimization [22]. Similar findings were also available among studies that were focusing on school-age adolescents in Hong Kong [23] and Macau [24], two Special Administrative Regions of China. Additionally, some studies have delineated several explanatory mechanisms by which family violence is linked to youth violence, including genetic connection between violent parents and violent children, brain injury caused by physical abuse, and violence-induced traumas that are disruptive to the development of brain and skills such as emotional regulation [25,26]. Despite the abundance of studies investigating the violence and violent victimization among youth in mainland China, most studies considered the overlap between violence and violent victimization resulted from victim lifestyle [23,27]. Such studies have vaguely indicated that victims were responsible for their victimization. Relatively few studies have considered crime victimization and violence as great public health issues. Moreover, a great number of studies only emphasized one aspect of ecological influence (e.g., family systems or peer systems) on either adolescent violent offending or victimization [28,29].

Therefore, the current study set out to explore whether Chinese adolescents who experienced high levels of legitimate violence in family spheres may engage in more culturally unacceptable violent behavior in society, and whether committing violence and violent victimization overlapped to some extent. If so, how does family violence lead to juvenile violence through the spillover mechanism? To the best of our knowledge, this study is the first study that systematically assessed family systems, violent norms, and violent peer association as three domains of the social environment that influence both adolescent violent offending and victimization among Chinese adolescents by using a longitudinal sample. By adding a focus on the role of peer relationship in the development of violent behavior and victimization, this study also presents an extension to the cultural spillover theory. Most studies that adopted the cultural spiller theory only examined the factors in the family system [30,31]. As informed by criminological research, peer relationship is closely connected to adolescent experiences at home and may play a critical role mediating the influences of the family system on adolescent violent behavior and victimization. By integrating peer association into the model, the current study can develop a more comprehensive understanding of the spillover effect of family processes on adolescent violence and victimization. 

## 2. Theoretical Background

### 2.1. Cultural Spillover Theory: Family Violence as a Predictor of Adolescent Violent Behavior

According to cultural spillover theory, culturally legitimate violence in one sphere of life may spill over into culturally unaccepted violence in other spheres of life. In other words, given the fact that the spill-over process transcends the confines of legitimate and illegitimate violence, people who receive continuing violence exposure, including culturally legitimate violence and culturally unaccepted violence, are more likely to resort to illegitimate violence to solve problems in their lives [32,33]. Children who are exposed to family violence are more likely to commit violence to get others to do what they want because a violent family culture develops the learning of violent behavior, and violence is normalized in the family culture. More recently, research has shown that culturally legitimate violence in the family system including the marital subsystem and the parent-child subsystem may engender violence in other social systems, including the peer system. A consistent amount of literature has demonstrated that witnessing interparental violence (IPV) or experiencing physical violence as a child or an adolescent in the family system may be linked to subsequent violence. 

Growing evidence indicates that witnessing interparental violence (IPV) as a child or an adolescent has been found to be associated with subsequent externalizing and internalizing behavior problems in later life [5,6,34,35]. For instance, in a longitudinal study of a representative sample, Romano et al. (2005) found that witnessing IPV was linked to aggressive oppositional children behavior [36]. In accordance with previous research, Renner and Boel-Studt demonstrated the association between witnessed IPV and internalizing and externalizing behaviors among children [11,12]. In a representative sample of 1050 children in South Africa, Cluver, Bowes, and Gardner demonstrated that witnessing IPV was linked to engagement in bullying [37]. A longitudinal study conducted by Baldry yielded similar results [38]. Along similar lines, Ireland and Smith found that exposure to IPV during childhood and adolescence led to violent crime in early adulthood [39]. Children who witness parental use of violence as a means to solve marital conflict may view violence as an acceptable way to resolve interpersonal disagreement and are inclined to turn to violence when confronted with a problem in their interactions with others. 

In addition to witnessing interparental violence (IPV), there is a consistent number of studies supporting that physical punishment and abuse are linked to internalizing and externalizing behaviors among children [11,12]. Children who experience physical violence in the parent-child dyad may view violence as a legitimate way to get others to do what one wants, and apply it in other contexts or relationships. In some families, physical punishment serves as legitimizing violence, so parents or other authority figures would impose it on children to shape child behavior and to ensure child conformity. The violent behavior would spill over from the parent-child relationship to other relationships in the social context. Support for the influence of physical punishment on juvenile violence was found in samples of 411 families across three generations, where Smith and Farrington demonstrated that harsh punishment led to the emergence of child violence [40]. Along similar lines, Gorman-Smith et al. found that young males who had experienced physical punishment were more likely to engage in dating violence than young males who had experienced no physical violence [41]. Similar results were also reported by Morris et al. in 2015 and other studies [42]. 

Taken together, when compared to other adolescents, adolescents who grow up with violence that is embedded in the family ecology are more likely to become a perpetrator of violent crime [43]. In the cascade of violence, adolescents may interpret the world in distorted ways where violence is a normal and efficient way to satisfy their burning needs [44]. 

### 2.2. The Mediating Role of Violent Norms and Violent Peers

As children enter into adolescence, they develop more complex relationships not only in a family system, but in different social systems. In puberty, friends and peers enact increasingly important roles in adolescent lives, especially for those adolescents who live in a hostile family environment [45,46]. The main reason lies in the fact that children who grow up with violent family dynamics may learn aggressive tactics to initially solve problems at home. In turn, such aggressive tactics would be carried over to other social systems, including peer relationships through the spillover mechanism. Under the influence of violence, children are more likely to display problem behaviors. Furthermore, they are unable to learn conflict resolution through communication and compromise. Such externalizing aversive interpersonal behaviors, social maladjustment coupled with violent norms lead the adolescents to be rejected by prosocial peers, further resulting in violent peer association. Furthermore, children’s aggressive behavior may cement friendships with other aggressive peers, which in turn reinforces the learning of violence and development of violent behavior [47].

Violent peer group membership serves multiple functions in finding support and regaining a sense of belonging to those adolescents exposed to uncertain and violent family environments [13]. Peer influence may serve as one of the explanations of the relationship between family violence and juvenile violence. For example, Makin-Byrd, Bierman, and the Conduct Problems Prevention Research Group (2013) demonstrated that exposure to family violence in childhood and early adolescence led to violence perpetration in late adolescence [14]. Moreover, violent peer association may also increase the likelihood of adolescent violent victimization as violent peer association leads to more exposure to violent behaviors, which heightens their vulnerability to violent victimization [48,49]. All of these studies suggest that peer influence may serve as one of the explanations of the relationship between family violence, youth violence, and victimization. Delinquent peers seem to be a critical link between family violence, youth violence, and victimization. Therefore, another objective of this study was to examine whether delinquent peers mediated the effect of family violence on youth violence and victimization.

### 2.3. The Current Study

**Figure 1:**
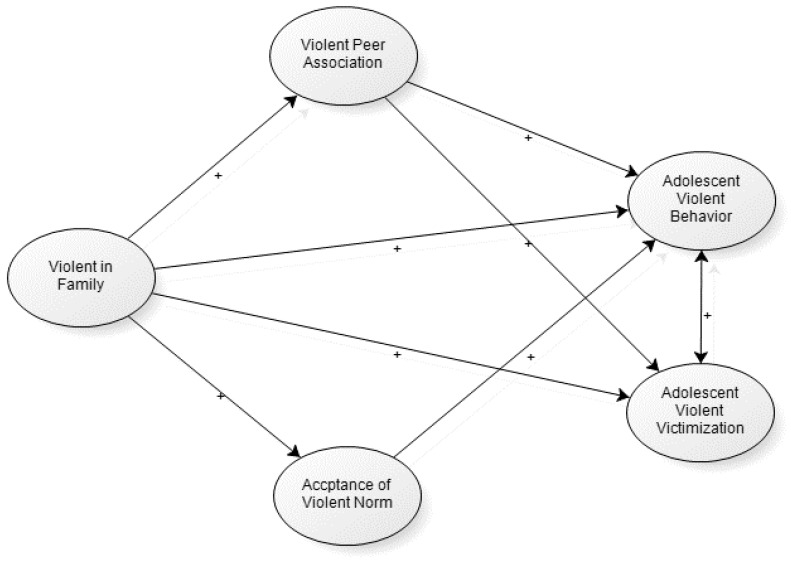
Theoretical framework.

To fill the gap in the literature, the objective of this study was to explore how family violence during childhood led to violence perpetration and victimization in adolescence through the cultural spillover mechanism among Chinese youths. Specifically, this study set the following main objectives (See Figure 1. for further information):

(1) To explore whether exposure to family violence during childhood led to violence perpetration and victimization in adolescence.

(2) To explore whether exposure to family violence during childhood strengthened violent norms due to the cultural spillover effect. In other words, we hypothesized that exposure to family violence during childhood would be positively related to violent norms in adolescence.

(3) To explore whether exposure to family violence during childhood led to violence perpetration in adolescence through violent norms.

(4) To explore whether exposure to family violence during childhood strengthened violent peer association due to the cultural spillover effect. In other words, we hypothesized that exposure to family violence during childhood would be positively related to violent peer association in adolescence.

(5) To explore whether exposure to family violence during childhood led to violence perpetration and victimization in adolescence through its association with delinquent peer association by the cultural spillover mechanism.

(6) To examine to what extent violent offending and violent victimization overlapped among Chinese adolescents.

## 3. Methods

### 3.1. Sample and Data

The data of this study is derived from the research project funded by University of Macau. The project identification code is MYRG2014-00120-FSS. The Panel on Research Ethics at the University of Macau has now given its approval for the project on December 19th in 2014. 

Researchers collect the data from one of the largest cities in Southwest China in 2015. Applying multi-stage stratified probability proportionate to size (PPS) sampling, the current study collected a total of 2384 middle school and high school students. Specifically, a list of all the school information was first obtained via the local municipal Department of Education. Districts within the city and schools within the selected district were then selected through PPS sampling. Finally, the students were randomly selected from the selected schools. The overall participation rate in the first wave of the study exceeded 95%. The missing cases were largely due to students’ absence from the schools on the date of survey. 

The same classes of students were surveyed the following year. The records of student information were linked through their full name, gender, and date of birth. However, due to the policies of the surveyed schools (e.g., the separation of science and arts, restructure of the classes, etc.), some classes were reorganized and some students could not be tracked in the following survey year. Thus, at last, 1192 students were successfully linked with a link rate of 50.0%. The first and second wave of the study are denoted as T1 and T2 respectively.

A structured, self-administrated questionnaire was used to collect information from the surveyed students. Trained researchers administered the survey in the classrooms. To prevent potential interference, we asked the class teacher to step out of the classroom when conducting the survey.

### 3.2. Measures

Violent behavior was measured by the sum of nine dichotomized items about whether the respondents had conducted any of the violent behaviors in the last year, including threatened to rob somebody, was involved in a gang fight, threatening others with a weapon, injuring others with a weapon, fight with others, threaten to beat somebody up, seriously injure someone, rob somebody with violence, and had sex with others against their will. Similarly, violence victimization was measured by five items about whether the respondents had been robbed, threatened with weapons, been injured with weapons, been beaten (slapped, choked, kicked), or been severely injured during the last year. 

Violence in the family system consisted of inter-parental violence and parent-to-child violence. The former was measured by two questions asking the respondents’ observations on how often father (mother) beat mother (father), the latter was measured by four questions asking the respondents how often their mother (father) beat/slap them. The response scale ranged from 1 = never to 5 = always.

Association with violent peers was measured by three items on the respondents’ perception of the proportion of friends who had been involved in fighting, threatening to use violence, and participating in gang fighting. The answer scale ranged from 1 = none to 5 = all. The Cronbach’s alpha value of these three items was 0.81, indicating good internal consistency. 

Violent norm acceptance was measured by four items. The respondents were asked whether they agreed that “escape fighting is cowardice”, “it is justifiable to fight back”, “fight is the only way to respond when teased”, and “my friend think I am afraid if I refuse to fight”. The answer scale ranged from 1 = strongly disagree to 5 = strongly agree. A higher value indicated a higher acceptance of the violent norm. The Cronbach's alpha value of these four items was 0.82.

Both violent and victimization behavior were measured in the first and second waves of the study, violence in the family system was measured in the first wave of the study, and the association with violent friends and acceptance of violent norm were measured in the second wave of the study. Age and gender were included as the demographic variables.

### 3.3. Analytical Approach

A descriptive analysis of all the variables that were used in the current study was first conducted to provide an overview of the current sample. Then, structural equation model (SEM) analysis was conducted to test the hypothesizes of the current study. The direct, indirect, and total effects of the studied variables with standardized coefficients were also presented. STATA 14.1 [50] was used to perform all the statistical analysis. The alpha was set to 0.05 throughout the study. The missing data was imputed via the mlmv (Maximum Likelihood with Missing Value) function in the STATA 14.1 SEM package [50].

## 4. Results

### 4.1. Sample Descriptive

Table 1 provides the descriptive analysis for all of the studied variables. Of the 1192 studied students, 50% were female, and the average age was 14.16. On average, the surveyed students had experienced little violence from their parents and violence also seldom occurred between their mother and father. In terms of the violence peer association, the respondents were exposed to a minor level of violent peers: the average scores of the prevalence peer were less than 2 = A little. Among the three types of associated violent peers, peers who were usually involved in fighting was the most prevalent type. In terms of acceptance of violence norm, the respondent students generally disagreed on using violence to show bravery, to response to teasing, or to avoid cowardice. The respondents tended to slightly support the use violence to fight back. Finally, the surveyed students reported that they had conducted less than one type of violence in the first year, and the number slightly increased in the following year. Meanwhile, the students also reported that they experienced less than one type of violent victimization, and the number decreased in T2.

### 4.2. Results of Structural Equation Models

Table 2 shows the correlation matrix of the variables to be used in the SEM, from which the bivariate relationships could be explored. The results in the lower left of the table present the correlations between violence in the family system to violent behavior and violent victimization both in T1 and T2. All of the correlation coefficients were positive and most of them were significant at *p* < 0.05 level, but smaller in size in T2 than in T1. These results partially support the first hypothesis of this study, which predicts a positive correlation between violence in the family system and violent/victimization. 

The correlation coefficients between violence in the peer system and each of the indicators measuring violence in the family system were also positive and significant at the *p* < 0.05 level. These results lend support to the second hypothesis of the cultural spillover hypothesis, which indicates that violence in the family system strengthened delinquent peer association.

Violence in the family system was also found to be significantly correlated with the acceptance of violent norm. The correlation coefficients between six items of violence in the family system and four items of violence acceptance reached significance at the *p* < 0.05 level. The results produced evidence of the third hypothesis of cultural spillover hypothesis.

Finally, the lower right of the tale describes the correlation between violence and victimization, and the same measurements across different time points. Consistent with our expectation, both violence and violent victimization in T1 predicted violence and victimization in T2 (r1 = 0.27, *p* < 0.05; r2 = 0.27, *p* < 0.05). The correlations between violence and victimization were also significant in both T1 and T2 (r1 = 0.33, *p* < 0.05; r2 = 0.29, *p* < 0.05), indicating that violence and victimization significantly overlapped.

**Figure 2:**
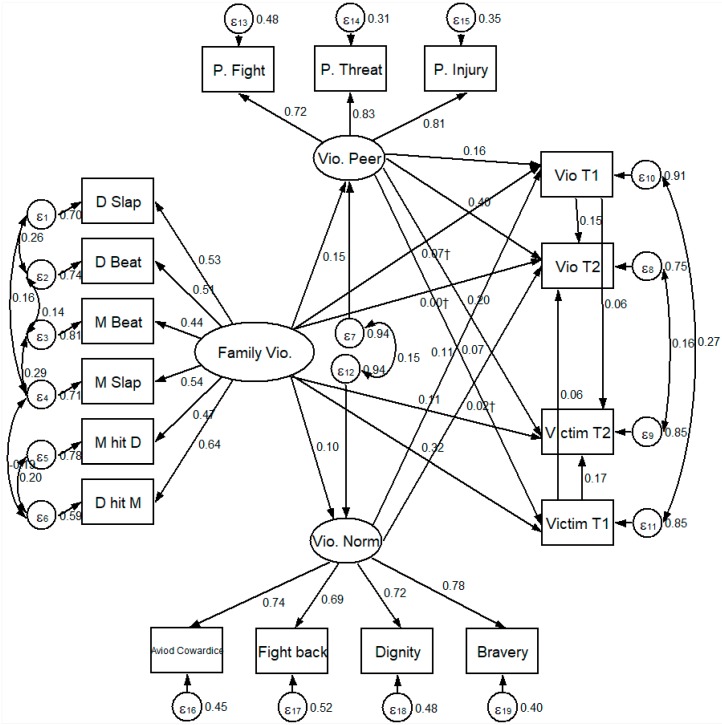
X^2^ = 297.561, d.f. = 119; Root Mean Square Error of Approximation, RMSEA = 0.035; Comparative Fit Index, CFI = 0.968; Tucker–Lewis Index, TLI = 0.955. All of the coefficients are standardized. The model controls gender and age. D and M stand for dad and mom, respectively. All coefficients reached significance at the *p* < 0.05 level except for those with †.

The bivariate correlations in Table 2 lend support to the first to the third research hypothesizes. SEM was then conducted to investigate whether the results in the bivariate analysis were robust against the control variables. Figure 2 displays the detail setting of the SEM. Generally speaking, the SEM model was a good fit (X2 = 297.561, d.f. = 119; Root Mean Square Error of Approximation, RMSEA = 0.035; Comparative Fit Index, CFI = 0.968; Tucker–Lewis Index TLI = 0.955). All of the goodness of fit indexes reached acceptable levels. 

The factor loadings of the six indicators of violence in the family system, three indicators of violent peers, and four indicators of violent norm all exceeded 0.45, indicating that all the indicators could be considered as proper measurements to the relative latent concepts. Suggested by the Modification Index, some error terms were allowed correlated. 

In the structural part, violence in the family system was positively related to violence in the peer system (β = 0.15, *p* < 0.05) and violent norms (β = 0.10, *p* < 0.05), which further supported the second and the third hypothesis. Violence in the family system was positively and directly related to violent victimization both in T1 (β = 0.32, *p* < 0.05) and T2 (β = 0.11, *p* < 0.05), but no significant direct effect was found between violence in the family system and violence. The direct effect of the violence in the peer system was significantly and positively related to violence (β_T1_ = 0.40, *p* < 0.05; β_T2_ = 0.16, *p* < 0.05) and violent victimization (β_T1_ = 0.20, *p* < 0.05; β_T2_ = 0.07, *p* < 0.05). Violent norm was found to have a significant effect on violent behavior only in T1 (β = 0.07, *p* < 0.05), but not in T2.

Strong cross-time effects were also found on violence and victimization in both T1 and T2. Violence in T1 predicted violence in T2 (β = 0.15, *p* < 0.05) and violent victimization in T2 (β = 0.06, *p* < 0.05). Similarly, violent victimization in T1 predicted both violence in T2 (β = 0.06, *p* < 0.05) and violent victimization in T2 (β = 0.17, *p* < 0.05). 

As the SEM figure only allowed us to observe the direct effects, Table 3 further calculated the indirect and total effects of each exogenous variable to investigate the fourth and fifth hypotheses. Violence in the family system, despite having no significant direct effect on violent behavior both in T1 and T2, indirectly fostered violence through increasing violent peer association and the acceptance of violent norms. On the other hand, violence in the family system had both direct and indirect effects on violent victimization. It is likely that violence in the family system is one of the sources of violent victimization. Violence in the peer system directly affected both violence and victimization in T1 and T2. When comparing the total effects of violence in the family system and peer system, it is likely that violence in the peer system has a higher effect on individual’s violent behavior, while violence in the family system has a higher effect on violence victimization. The result can be interpreted as the different roles that violence in the family and peer system play on the individuals’ violent behavior and violent victimization. Violent norm had a significant effect only on the violent behavior in T1, its effect on violent behavior in T2 was exclusively indirect.

## 5. Discussion

Family violence on children including physical punishments and witnessing IPV, is one of the most pervasive public health problems in the world. Family violence may not only inflict appalling bodily injuries, but also cast an unfading shadow on children wellbeing. It may further result in violence perpetration or victimization in later stages of life. In other words, family violence begets more violence, which thrusts children into a vicious cycle of violence. In China, the proverb “spare the rod and spoil the child” serves as a justification for using physical punishments in child rearing, thus family violence on children serves as legitimate violence in China. According to cultural spillover theory, a family culture with a high level of legitimate violence may contribute to illegitimate violence in other relationships in social contexts [32]. Based on the theory, the purpose of the current study was to determine whether growing up in violent family contexts increased the risk of violence among Chinese adolescents through violent norms and the association with violent peers by the cultural spillover mechanism. The current study examined both the experience of parental violence and of witnessing IPV as potential risk factors for violence perpetration and victimization. One mediating factor was the violent norm, and the other factor was violent peer association based on existing literature concerning youth violence. 

This study extended the previous knowledge of the cultural spillover theory in several ways by exploring the causal effect of these variables during the child developmental period from childhood to early adolescence. First, the results demonstrated that exposure to family violence during childhood could spill over into other forms of violence in early adolescence through delinquent peer association among Chinese adolescents. The findings supported the notion that exposure to family violence, including parental violence or witnessing IPV, may result in a continuing cycle of violence in the family or other social systems [32]. However, it is worth noting that exposure to family violence during childhood does not directly lead to violence in early adolescence. Rather, violent norms and violent peer association mediate the effect on the relationship from family violence to violence perpetration in later stages of life among the participants in the current study. Our results were consistent with prior studies that suggested that adolescents who grow up in a violent family culture may develop learnt violence, and that such learning would be reinforced because of violent peer association. Violent peer association was identified as a stronger risk factor for violence preparation than family violence [51]. Living with family violence makes adolescents vulnerable to delinquent peer influence, which in turn leads to engagement in violent behavior in later stages of life.

Second, the results of the current study presented evidence that exposure to family violence during childhood predicted victimization in early adolescence. As expected, violent peer association partially mediated the relationship between family violence and violence victimization. A growing consensus has emerged that experiencing family violence during childhood contributes to violent victimization in later stages of life. Children who suffer family violence may display internalizing behavior, which in turn leads to becoming socially withdrawn [52]. These children, when compared to their peers, are more likely to become violent victims at school or in the workplace [53]. Additionally, a substantial body of research has suggested that receiving harsh punishment during childhood increases the risk of violent victimization [42]. Along similar lines, research has found that witnessing IPV as children are more likely to fall victims to violence in later peer relationships [5,51].

Finally, these results suggest that there were overlaps between violence and victimization. The results demonstrated that violence perpetration would lead to future victimization, and prior victimization was linked to violence perpetration after controlling other variables, including family violence, delinquent peer association, and other variables. The cycle of violent events continually repeats itself. As expected, cultural spillover theory alone would predict the overlaps between violence perpetration and victimization. Adolescents who grow up with a culture of violence would identify violence as an efficient way to solve their problems. Embedded with such a violent culture, violence perpetration may make violent adolescents vulnerable to violent victimization, as violent retaliation is a favorable response to one’s own victimization [33]. 

According to the findings in this study, suffering family violence during childhood was significantly related to violent victimization in early adolescence. Furthermore, experiencing violent victimization during childhood significantly increased the likelihood of violence perpetration in early adolescence. One of the main reasons lie in the fact that violent peer groups may provide support and a sense of belonging to those adolescents who grow up with family violence. However, violent peer association may lead those children to fall into other violence traps in their lives. For example, De La Ruem and Espelage found that adolescents who suffered sexual abuse or other forms of violence at home reported more gang involvement than their peers [54]. Family violence coupled with delinquent peer association would lead to subsequent violence perpetration and victimization. Therefore, it is necessary to work with families to prevent violence within the family context to break the cycle of violence. In the first place, it is imperative to implement primary preventions to end physical punishment in child rearing. Chinese parents should be aware that physical punishment is one form of family violence. The cumulative number of countries that have prohibited physical punishment on children as of 2017 is more than 50 countries [55]. Accordingly, the study suggested that the authorities could prohibit physical punishment on children to not only maintain children’s physical and mental health, but also to prevent future violence perpetration and victimization. Moreover, parents have to know that committing intimate partner violence has a negative impact on children. With regard to execution, the authorities have to work with communities to provide information relating to family violence.

This study calls for legislation to work in cooperation between communities, education, and awareness campaigns, schools, and government agencies to provide prevention and interventions against family violence. Furthermore, schools play essential roles in the prevention of future violence perpetration and victimization in several domains. First, school teachers have to participate in training programs designed to address family violence. Second, school teachers have to identify children who are at high risk of family violence. After identification, the teachers need to cooperate with government agencies to provide therapeutic intervention for those children who have experienced family violence. Meanwhile, government agencies have to provide immediate intervention to individuals who commit family violence to minimize the risk of family violence. Third, schools could serve as agents that provide support for children who suffer family violence. Schools could help develop positive social bonds between prosocial peers and those children, which in turn, would reduce the risk of delinquent peer association, thus also preventing future violence perpetration and victimization [56]. 

Although the study made substantial contributions to research and policy implication, there are two major limitations of this study. Notwithstanding all of the team members who put in a great deal of effort to collect a representative sample of youth participants, participants were lost in the follow-up. The reason lies in the fact that classes were reorganized after a year, so some participants were not followed up during the second year. Furthermore, the sample was regionally representative rather than nationally representative, so the findings were limited to a generalization of the national adolescent population. Therefore, a planned time series analysis of nationally representative data is required for a future study. 

## 6. Conclusions

In conclusion, as postulated by the hypothesis in this study, the study found that adolescents who had suffered family violence during childhood were more likely to be exposed to violent peer influence and to accept violent norms, which increased the likelihood of violence perpetration in early adolescence. Furthermore, family violence including the direct experience of violence from a parent, and witnessing IPV, led to future violent victimization. Finally, the study demonstrated that there were overlaps between violence perpetration and victimization. The study also provides related suggestions on policy implications to break the cycle of violence.

## Figures and Tables

**Table 1:** Descriptive analysis of studied variables.

Variables (*N* = 1192)	Mean	S.D.	Min	Max
Female	0.50	0.50	0	1
Age	14.16	1.73	10	18
Dad slapped you	1.96	1.14	1	5
Dad beat you	1.32	0.75	1	5
Mom slapped you	1.37	0.84	1	5
Mom beat you	1.83	1.13	1	5
Mom hit Dad	1.47	0.81	1	5
Dad hit Mom	1.47	0.86	1	5
Association with Violent Peers (Fighting)	1.55	0.81	1	5
Association with Violent Peers (Threating)	1.29	0.66	1	5
Association with Violent Peers (Beating to a pulp)	1.20	0.58	1	5
Violent norms (Fighting to avoid cowardice)	2.28	1.18	1	5
Violent norms (Being justifiable to fight back)	3.06	1.30	1	5
Violent norms (Fighting when being teased)	2.45	1.28	1	5
Violent norms (Fighting to show bravery)	2.63	1.35	1	5
Violence T1	0.38	0.75	0	9
Violence T2	0.40	1.12	0	9
Victim T1	0.71	0.95	0	5
Victim T2	0.54	0.96	0	5

The first and second wave of the study are denoted as T1 and T2 respectively.

**Table 2:** Correlation coefficients matrix of related variables.

	Variables	(1)	(2)	(3)	(4)	(5)	(6)	(7)	(8)	(9)	(10)	(11)	(12)	(13)	(14)	(15)	(16)	(17)
**(1)**	Dad slap you	1.00																
**(2)**	Dad beat you	0.44	1.00															
**(3)**	Mom slap you	0.22	0.33	1.00														
**(4)**	Mom beat you	0.38	0.24	0.46	1.00													
**(5)**	Mom hit Dad	0.23	0.24	0.30	0.29	1.00												
**(6)**	Dad hit Mom	0.36	0.37	0.28	0.23	0.44	1.00											
**(7)**	Assoc. with Vio. Peers (Fighting)	0.13	0.05	0.06	0.14	0.12	0.10	1.00										
**(8)**	Assoc. with Vio. Peers (Threating)	0.13	0.06	0.06	0.12	0.08	0.09	0.59	1.00									
**(9)**	Assoc. with Vio. Peers (Beating to a pulp)	0.09	0.06	0.08	0.12	0.09	0.10	0.58	0.69	1.00								
**(10)**	Violent norms (Fighting to avoid cowardice)	0.02	–0.02†	0.04	0.03	0.06	0.04	0.15	0.12	0.15	1.00							
**(11)**	Violent norms (Being justifiable to fight back)	0.05	0.04	0.01†	0.05	0.02	0.04	0.11	0.10	0.06	0.50	1.00						
**(12)**	Violent norms (Fighting when teased)	0.09	0.04	0.07	0.10	0.03	0.09	0.10	0.13	0.11	0.52	0.54	1.00					
**(13)**	Violent norms (Fighting to show bravery)	0.07	0.00†	0.05	0.04	0.04	0.05	0.16	0.09	0.13	0.60	0.53	0.54	1.00				
**(14)**	Violence T1	0.12	0.04	0.05	0.07	0.04	0.05	0.22	0.15	0.13	0.12	0.12	0.09	0.13	1.00			
**(15)**	Violence T2	0.05	0.00†	0.10	0.06	0.11	0.08	0.39	0.35	0.36	0.16	0.08	0.07	0.13	0.27	1.00		
**(16)**	Victim T1	0.24	0.15	0.17	0.22	0.16	0.20	0.17	0.15	0.11	0.08	0.12	0.10	0.11	0.33	0.19	1.00	
**(17)**	Victim T2	0.16	0.13	0.10	0.14	0.07	0.10	0.24	0.25	0.18	0.10	0.14	0.11	0.14	0.18	0.29	0.27	1.00

Note: All the correlation coefficients reach *p* < 0.05 level except those coefficients with †.

**Table 3:** Direct, indirect, and total effects of family violence, peer violence.

Outcomes	Family Violence	Violent Peer	Violent Norm	Victim T1	Violence T1
Violence T1					
Direct	0.08 (0.07)	0.21 (0.16) ***	0.06 (0.07) *	--	--
Indirect	0.04 (0.03) **	--	--	--	--
Total	0.12 (0.10) **	0.21 (0.16) ***	0.06 (0.07) *	--	--
Violence T2					
Direct	0.00 (0.00)	0.77 (0.40) ***	0.02 (0.02)	0.07 (0.06) *	0.22 (0.15) ***
Indirect	0.18 (0.10) ***	0.06 (0.03) ***	0.01 (0.01) *	--	--
Total	0.18 (0.10) ***	0.83 (0.43) ***	0.04 (0.03)	0.07 (0.06) *	0.22 (0.15) ***
Victim T1					
Direct	0.50 (0.32) ***	0.18 (0.11) ***	--	--	--
Indirect	0.03 (0.02) **	--	--	--	--
Total	0.53 (0.34) ***	0.18 (0.11) ***	--	--	--
Victim T2					
Direct	0.18 (0.11) **	0.33 (0.20) ***	--	0.17 (0.17) ***	0.07 (0.06) *
Indirect	0.15 (0.10) ***	0.05 (0.03) ***	--	--	--
Total	0.33 (0.21) ***	0.38 (0.23) ***	--	0.17 (0.17) ***	0.07 (0.06) *

^1^ * *p* < 0.05; ** *p* < 0.01; *** *p* < 0.001. Standardized coefficients are in the parenthesis.
